# ASO Author Reflections: Comparison of Indocyanine Green with Blue Dye in Sentinel Lymph Node Biopsy for Cutaneous Melanoma

**DOI:** 10.1245/s10434-023-13423-5

**Published:** 2023-04-04

**Authors:** Michael G. Fadel, Myles J. Smith

**Affiliations:** The Sarcoma, Melanoma and Rare Tumours Unit, The Royal Marsden Hospital and Institute Cancer of Research, London, United Kingdom

## Past

The use of dual modalities to identify the sentinel lymph node (SLN) in cutaneous melanoma improves the detection rate and sensitivity of the test.^1^ However, commonly used blue dyes may have several drawbacks including discoloration of the skin locally and in the target lymph node bed during dissection. Although rare, they can also cause the patient to have a change in urine color and a postoperative pallor. Of most concern is the risk of perioperative anaphylactic reaction associated with blue dye, which can be in the region of 1% in patients.^2^ This is a serious, potentially life-threatening event and thus causes concern for the surgeon, anesthetist, and perioperative medicine providers. Therefore, if dual modalities for identifying the SLN are used, an agent that potentially improves visualization and safety, reducing the risk of anaphylactic reactions, would be preferable.

Within this context, we sought to investigate the use of indocyanine green (ICG) near-infrared imaging as a modality for SLN biopsy in addition to single photon emission computed tomography/computed tomography (SPECT-CT). We aimed to compare ICG with blue dye for the identification of the SLN and evaluate any benefits that ICG may have for the patient or surgeon longitudinally to plan for future studies.

## Present

The study demonstrated that using ICG near-infrared imaging in addition to SPECT-CT was not inferior to blue dye for the identification of the SLN in melanoma within the context of our institution and practice.^3^ Of the 285 patients that underwent wide local excision and SLN biopsy, 122 patients received ICG and 163 patients had blue dye. ICG had a higher SLN detection rate (96.7% vs. 89.6%, *p* = 0.022) and equal pathological SLN detection rate (92.3% vs. 97.1%, *p* = 0.481) when compared with blue dye. There were no differences between ICG and blue dye in terms of postoperative complications, including seroma rates. Furthermore, we reported no cases of allergic reaction or anaphylaxis with the use of either ICG or blue dye.

We found potential advantages to using ICG and near-infrared imaging including the ability to see through tissue to identify lymphatics and the SLN, its specificity after the node has been removed and the fact that there is no apparent discoloration of the skin, urine or general appearance of the patient. Ultimately, we observed that the time spent performing SLN biopsy reduced for individual surgeons over time, and anecdotally, that it was particularly useful as an adjunct in a challenging deep axilla. However, there is an inherent learning curve present and in certain instances, such as head and neck, we did not find ICG to be as useful when compared with extremity cases due to oversaturation of the target node and surrounding tissue. Moreover, the capital investment required is significant if one is to invest in near-infrared imaging for SLN biopsy alone.

## Future

In our center, most surgeons performing SLN biopsy for melanoma now use near-infrared imaging with ICG due to the advantages we perceived and have presented. As this technology becomes more integrated into surgical use, and into laparoscopic stacks, there will be greater opportunity for application as centers continue to upgrade their equipment. The exact role of fluorescence-guided surgery in melanoma should be assessed in future studies to establish the utility of ICG more definitively in SLN biopsy and lymphatic and nodal mapping for advanced melanoma. There may also be the possibility of ICG providing transdermal visualization in guiding interval nodes in certain cases in which the nodal basin is predictable. Beyond that, there is the potential for comparison of different techniques, such as magnetic guidance and augmented reality guidance, to the current standard of care in SLN biopsy. However, the greatest potential for fluorescence-guided surgery in melanoma may relate to the development of tumor-specific probes and their capability for intraoperative guidance, which may allow us to reduce surgical morbidity and improve oncological outcomes in challenging and advanced disease.^4^
